# Neural Network-Based Granular Activity Recognition from Accelerometers: Assessing Generalizability Across Diverse Mobility Profiles

**DOI:** 10.3390/s26041320

**Published:** 2026-02-18

**Authors:** Metin Bicer, James Pope, Lynn Rochester, Silvia Del Din, Lisa Alcock

**Affiliations:** 1 Translational and Clinical Research Institute, Faculty of Medical Sciences, Newcastle University, Newcastle upon Tyne NE1 7RU, UK; metin.bicer@newcastle.ac.uk (M.B.); lynn.rochester@newcastle.ac.uk (L.R.); lisa.alcock@newcastle.ac.uk (L.A.); 2 NIHR Newcastle Biomedical Research Centre, Newcastle University and the Newcastle upon Tyne Hospitals NHS Foundation Trust, Newcastle upon Tyne NE4 5PL, UK; 3 School of Engineering Mathematics and Technology, University of Bristol, Bristol BS8 1QU, UK; jp16127@bristol.ac.uk

**Keywords:** human activity recognition, deep learning, wearable sensor

## Abstract

**Highlights:**

**What are the main findings?**

- Outperformed fixed sliding-window methods for frame-level activity recognition.
- Length of data window did not affect the performance.
- Satisfactory cross-cohort performance.

**What are the implications of the main findings?**

- Granular recognition for capturing transitions.
- Handling of long and arbitrary length real-world wearable sensor data.

**Abstract:**

Human activity recognition (HAR) lies at the core of digital healthcare applications that monitor different types of physical activity. Traditional HAR methods often struggle to adapt to variable-length, real-world activity data and to generalise across cohorts (e.g., from young to old cohorts). Thus, the aim of this study was to investigate HAR using wearable sensor data, with a particular focus on cross-cohort evaluation. Each dataset included two accelerometers (right thigh and lower back) sampling at 50 Hz, capturing a range of daily-life activities that were annotated using video recordings from chest-mounted cameras synchronised with the accelerometers. Neural networks were trained on young cohorts’ data and tested on old cohorts’ data. The effects of network architecture, sampling frequency and sensor location on classification performance were investigated. Network performance was evaluated using accuracy, recall, precision, F1-score and confusion matrices. The gated recurrent unit architecture achieved the best performance when trained solely on young cohorts’ data, with weighted F1-score of 0.95 ± 0.05 and 0.93 ± 0.05 for young and old cohorts, respectively, resulting in a highly generalizable method. Classification performance across multiple sampling frequencies was comparable. The thigh-mounted sensor consistently achieved higher performance than the lower back sensor across activities except lying. Furthermore, combining datasets significantly improved performance on the old cohort (weighted F1-score: 0.97 ± 0.02) due to increased variability in the training data. This study highlights the importance of network architecture and dataset composition in HAR and demonstrates the potential of neural networks for robust, real-world activity recognition across age-defined cohorts, specifically between young and old cohorts.

## 1. Introduction

Improving quality of life drives advances across domains, from smart homes [1] and industry [2] to healthcare [3], where physical mobility, a key indicator of health [4], is routinely evaluated through mobility assessment. Traditional mobility assessments lack ecological validity, but wearable sensors enable long-term monitoring in real-world environments [3]. This shift allows research to move from lab-based tests to daily-life monitoring, which rely on human activity recognition (HAR), including walking and sedentary behaviours [5,6,7]. Traditional HAR approaches often lack generalizability to old populations due to validation on small, homogeneous cohorts (typically 10–20 healthy adults) [7,8]. These methods relied on fixed thresholds and rule-based trees [9,10], which assume specific mobility characteristics, reducing applicability to diverse populations and increasing computational complexity. In contrast, data-driven HAR approaches detect activities directly from wearable signals, either with or without feature engineering. Feature-based methods extract time- and frequency-domain measures (e.g., mean, variance, entropy) [11], but lack established rules for selecting optimal features, particularly for activities with similar characteristics [12]. To overcome this challenge, researchers increasingly adopted neural networks [13]. Convolutional (CNNs) and recurrent (RNNs) neural networks have shown strong potential for HAR [14]. CNNs, adapted for time-series analysis, use 1D convolutional filters to extract spatial features [15,16,17], while RNNs capture temporal dependencies [18,19]. Hybrid CNN–RNN networks exploit both spatial and temporal information in sensor data [20,21]. More recently, architectures that model longer-range dependencies, such as temporal convolutional networks, self-attention mechanisms and transformer-based architectures, have also been applied to solve this problem [22,23,24].

Previous studies predicted a single activity label per 2–3 s window [25,26], which reduces granularity and can mislead training when multiple activities occur within a window [27]. HAR at a more granular level (i.e., dense prediction across the time series, where activity transitions are reflected by changes in the predicted label window rather than by assigning a single label to a multi-second window) remains underexplored [27,28]. Furthermore, existing HAR datasets differ in sensor modalities and placements, which complicates comparisons between studies [29,30]. Healthy old adults are also underrepresented in commonly used datasets [31,32,33], meaning that cross-cohort evaluation is not routinely performed, although it has been explored in recent work [34]. Sensor placement and the number of sensors influence both performance and usability: while multi-sensor configurations can improve classification accuracy, extensive setups may restrict natural movement and reduce wearability, particularly for old adults and healthcare applications. Sampling frequency presents a similar trade-off, as higher frequencies capture finer motion detail but increase power consumption and storage requirements, limiting feasibility for long-term monitoring in real-world applications. Consequently, optimising sensor placement and sampling frequency is essential to balance accuracy, usability, and real-world applicability.

This study aimed to develop and evaluate neural networks for recognising daily activities using publicly available datasets from young and old cohorts. We trained models on data from the young cohort and evaluated their performance on the old cohort to assess cross-cohort generalisability. The objectives were to (1) develop neural networks for HAR in the young cohort, (2) evaluate their performance in the old cohort, and (3) examine whether combining datasets from the young and old cohort improves model performance. To support these objectives, we investigated the influence of network architecture, sampling frequency, and sensor location. By focusing on frame-level recognition (i.e., predicting at each time step) and cross-cohort evaluation between the young and old cohorts, this work supports the development of practical HAR methods for real-world healthcare applications.

## 2. Materials and Methods

### 2.1. Datasets

This study utilised two publicly available HAR datasets [34,35], both containing tri-axial accelerometer data, sampled at 50 Hz, recorded from the lower back and the right thigh in ambulatory settings. Sensor orientation was consistent between studies, and no additional axis re-alignment was required. The accelerometer data were used in their raw form for training and evaluation, with no gravity removal or coordinate normalisation applied in this study. Both datasets used in this study provided activity labels for each frame, derived from synchronised video recordings, using the annotation protocols described in their respective publications [34,35]. In this work, we used these labels as provided and did not apply any additional post-processing methods. This was a deliberate choice to preserve reproducibility and to evaluate the robustness models under realistic annotation conditions, where some degree of ambiguity around activity transitions is unavoidable. Additionally, both datasets exhibit substantial class imbalance, with postural and ambulatory activities (e.g., sitting, standing, walking) dominating the data, and more challenging activities such as stair ascent, stair descent and cycling occurring relatively infrequently. No additional resampling, class balancing or label modification was applied in this study. Detailed quantitative distributions for each activity class are provided in the corresponding publications [34,35].

The first dataset, referred to as the “Young Cohort Dataset” [35], comprised data from 22 young participants (8 female, 14 male; age: 38.6 ± 14 years; height: 177.3 ± 8.3 cm; weight: 72.9 ± 10.6 kg; BMI: 23.1 ± 2.3 kg/m^2^). The per-person average duration of data collected from this cohort was 97.90 ± 32.95 min. This dataset included nine distinct activities: walking, running, standing, stair ascent, stair descent, sitting, lying, cycle-sit and cycle-stand. The second dataset, referred to as the “Old Cohort Dataset” [34], included data from 18 old adult participants (9 female, 8 male; age: 79.6 ± 7.6 years; height: 173.0 ± 7.8 cm; weight: 80.0 ± 9.3 kg; BMI: 26.8 ± 2.7 kg/m^2^). The per-person average duration of data for this cohort was 41.84 ± 5.77 min. This dataset contained a subset of activities more common in an old population: walking, stairs ascending, stairs descending, standing, sitting and lying. Further details about the datasets can be found in their respective articles.

For both datasets, each timepoint included six sensor channels: three axes for the lower back accelerometer and three axes for the right thigh accelerometer. A single training or testing example window was a 6×T matrix, where T denotes the number of timepoints (i.e., the window length) for that specific example. Each time window had corresponding labels (ground-truth) for every timepoint such that a single example of a label window is a vector of length T. The window length for training was fixed to T=200 frames.

### 2.2. Neural Network Architectures

A critical design principle for all investigated architectures was their ability to process variable-length input windows and to predict an activity at each individual timepoint within the given window. This approach was chosen to ensure high temporal granularity in activity detection while maintaining computational efficiency by eliminating use of data windowing.

All investigated architectures were explicitly designed to process variable-length input windows and to generate predictions at each timepoint. No zero padding was applied at the input level to equalise window lengths, as the networks operate natively on windows of arbitrary length. Hybrid architectures were evaluated to assess whether combining local feature extraction (CNN) with long-range temporal modelling (RNN) offers advantages over purely recurrent models for dense activity recognition.

#### 2.2.1. One-Dimensional Convolutional Neural Network

One-dimensional CNNs are a type of neural network architecture designed for analysing sequential data, where the temporal or spatial order of the input is meaningful. These networks operate by applying filters (also known as kernels) that slide over the input, enabling the network to automatically learn spatially localised patterns, such as characteristic features in time series data.

In our implementation, the input was a window of sensor data denoted as $X=[x_{1},\;x_{2},\;\ldots ,x_{T}]$ where T is the length of the window and each $x_{t}\in R^{n}$ was composed of n sensor channel readings at time t. A convolutional layer consisted of a set of kernels, each defined by a weight vector $W=[w_{1},\;w_{2},\ldots ,\;w_{k}]$ with kernel size k. Kernels also had an associated bias term b. The convolution operation between the kernel W and a data window of X, followed by a non-linear activation function (Rectified Linear Unit, ReLU), produced an output feature map o according to $o_{t}=ReLU\left(\sum_{i=1}^{k}w_{i}\cdot x_{t+i-1}+b\right)$ at timepoint t. The output calculation was repeated across the entire window and for multiple kernels, enabling the network to learn diverse feature representations. The sliding movement of the kernels across the input window was governed by a parameter called the stride (i.e., the step size by which the kernel moves).

The implemented CNN architecture consisted of a series of 1D convolutional layers, each followed by a dropout and batch normalisation layer. The number of layers (1–5), number of kernels per layer (32–1024), kernel and stride size (1–11), dropout rate (0.3, 0.6) and learning rate (0.001–0.1) were optimised through grid search as described in Section 2.3.

#### 2.2.2. Gated Recurrent Unit Network

RNNs are a class of neural architectures designed to model sequential data by maintaining a memory of previous inputs through internal hidden states. Traditional RNNs, however, are limited in modelling long-range dependencies due to issues such as vanishing or exploding gradients during training. To address these limitations, gated recurrent architectures such as Long Short-Term Memory (LSTM) networks and gated recurrent units (GRUs), as well as attention-augmented recurrent variants, have been proposed to improve temporal modelling. In preliminary experiments, we evaluated vanilla RNN, LSTM, and GRU architectures, including attention-augmented variants. As the standard GRU architecture provided improved performance with more stable training behaviour and lower computational cost, it was selected as the recurrent architecture for subsequent experiments. GRUs regulate information flow through gating mechanisms that dynamically control the retention and updating of temporal information.

In our implementation, at each time step t, a GRU cell received the current input $x_{t}$ and the previous hidden state $h_{t-1}$. It then computed the new hidden state $h_{t}$ using two gates: the update gate $z_{t}=A(W_{z}x_{t}+U_{z}h_{t-1}+b_{z})$ and the reset gate $r_{t}=A(W_{r}x_{t}+U_{r}h_{t-1}+b_{r})$ where A denotes the activation function, $W_{*}$ and $U_{*}$ are learnable weight matrices and $b_{*}$ are bias terms. The update gate $z_{t}$ controls the extent to which the hidden state is updated with new information, while the reset gate $r_{t}$ determines how much of the past information to forget. Using these gates, the GRU computes a candidate hidden state ${\tilde{h}}_{t}=\;tanh\;[W_{h}x_{t}+U_{h}\left(r_{t}\cdot h_{t-1}\right)+b_{h}]$. The final hidden state is then updated by interpolation the previous hidden state and the candidate state, $h_{t}=\left(1-z_{t}\right)\cdot h_{t-1}+z_{t}\cdot {\tilde{h}}_{t}$. This gating mechanism allows the GRU to capture dependencies over longer windows without suffering from gradient degradation.

The implemented GRU architecture consisted of a series of recurrent layers, each followed by a dropout and batch normalisation layer. The number of layers (1–3), number of units per layer (32–256), dropout rate (0.3, 0.6) and learning rate (0.001–0.1) were optimised through grid search as described in Section 2.3.

#### 2.2.3. Hybrid Convolutional–Recurrent Networks

To leverage the strengths of both convolutional and recurrent neural networks in modelling sequential data, we implemented a hybrid architecture combining 1D CNNs with GRUs. One of the two architectures processes input window and its output is fed to the next architecture to output final predictions. We investigated two alternatives. The first hybrid architecture, called ConvRec, consisted of one or more layers of 1D CNNs processing the raw multivariate input window to extract local features. If strides greater than one were used, we applied padding to these features to retain input window length. Because the GRUs processed the full window while maintaining the input window length, the network retains the ability to generate a prediction for each individual time step. The second alternative, called ReConv, was to learn temporal features at the beginning with the GRU layers that was then followed by 1D convolutional layers to learn local features among the temporal features.

The hybrid networks were constructed by combining the CNN and GRU architectures as described in the previous section. The same hyperparameter search space was applied to the respective CNN and GRU components.

### 2.3. Training and Evaluation Strategy

Training of network requires optimising the kernel weights and biases. The optimisation problem was minimising the cross-entropy loss function quantifying the difference between the predicted probability distribution of the network and the true distribution of the data. The optimisation was carried out in PyTorch (V2.4.1) using a gradient-based optimisation method called adaptive moment estimation.

The Young Cohort Dataset was used for training neural networks (Objective 1) and initially divided into training, validation and test folds, following the approach described in [35], to tune hyperparameters. Hyperparameter tuning involved a random grid search with 50 configurations on several parameters (learning rate, number of convolutional or GRU layers, units, kernel and stride size, dropout rate) to optimise performance on the validation set, preventing overfitting to the training data. After the hyperparameter tuning, a Leave-One-Participant-Out Cross-Validation (LOPO-CV) strategy was employed to comprehensively assess the networks’ generalizability within the young cohort. In this strategy, for each fold, data from one participant was held out as the test set, while the remaining participants’ data were used for training. This process was repeated 22 times, ensuring that each participant’s data served as the unseen test set once. The predictions and true labels for all 22 individuals were then concatenated into single arrays to compute overall evaluation metrics for the young cohort. This approach provides a robust measure of generalizability at the individual participant level.

The Old Cohort Dataset was reserved for final testing to assess generalizability to an unseen population (Objective 2). Data from each of the 18 participants in the Older Cohort Dataset was passed through all 22 trained networks from the LOPO-CV. This resulted in 22 individual activity predictions at each timepoint for every participant. To obtain a final prediction for each timepoint, we applied a majority voting scheme: the most frequently predicted activity among the 22 networks was selected as the final activity label. In the event of a tie, the class with the highest summed prediction probability across networks was selected. These final predictions and their corresponding true labels were combined into single arrays to calculate overall evaluation metrics for the old cohort. This rigorous testing methodology ensured an unbiased and comprehensive assessment of the real-world generalizability.

We run additional experiments on sampling rate and sensor locations. First, a set of experiments was conducted in which the dataset was independently downsampled to 25, 20, 15, and 10 Hz, and model performance was evaluated at each sampling frequency to assess the effect of temporal resolution. Next, effects of sensor location were investigated by training networks using thigh-only, lower-back-only and combined sensor configurations. These experiments were carried out using the same strategy outlined above for the architecture selection.

We also developed a general neural network, based on the best-performing architecture, sampling frequency and sensor location, using a combined dataset by pooling all samples from the two cohorts (Objective 3). This neural network was evaluated using the same LOPO-CV strategy. To assess the impact of dataset composition, performance was evaluated separately for young and old participants. This allowed us to quantify how combining datasets influenced performance across the two cohorts.

We evaluated the performance of the trained models under different window lengths, specifically comparing 200-frame windows (4 s) with extended 3000-frame windows (1 min).

### 2.4. Performance Metrics

The performance of the trained networks was quantitatively assessed using several common classification metrics: precision, recall, accuracy and F1-score. These metrics were calculated both for each individual activity class (per-activity) and as macro averages across all activities. To account for imbalances in the number of samples per activity type, weighted averages of these metrics were also computed, using the proportion of samples for each activity as weights. Weighted F1-score served as the criteria for hyperparameter tuning. Also, this metric was used to compare the performance of models based on architecture, sampling frequency and sensor location. Performance differences between experimental conditions were assessed using paired t-tests on F1-scores, with statistical significance defined at *p* < 0.05.

A detailed breakdown of the networks’ performance was provided through the analysis of confusion matrices. A confusion matrix visually summarises the performance of a classification algorithm. The diagonal elements of the matrix represent the number (or percentage) of correct predictions for each activity class, where the predicted label perfectly matches the actual ground truth label. The off-diagonal elements indicate misclassifications, showing instances where one activity class was incorrectly predicted as another. Analysing the confusion matrix is crucial for identifying specific patterns of confusion between different classes and for understanding a network’s strengths and weaknesses for each activity.

## 3. Results

### 3.1. Neural Network Architecture Validation on the Young Cohort Dataset and Generalizability to the Old Cohort Dataset

**Figure 1:**
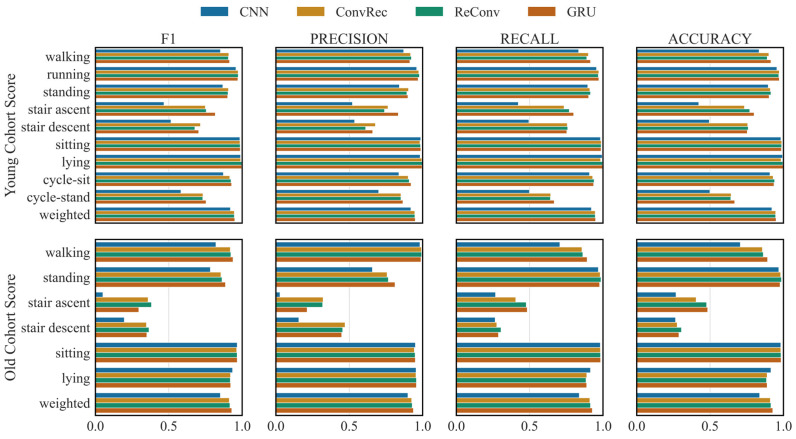
F1-score, precision, recall and accuracy on the Young and Old Cohort Datasets for different architectures. CNN: convolutional neural network. GRU: gated recurrent unit. ConvRec: CNN followed by GRU. ReConv: GRU followed by CNN. The metrics per activity and their average weighted by their frequency (“weighted”) are presented.

**Figure 2:**
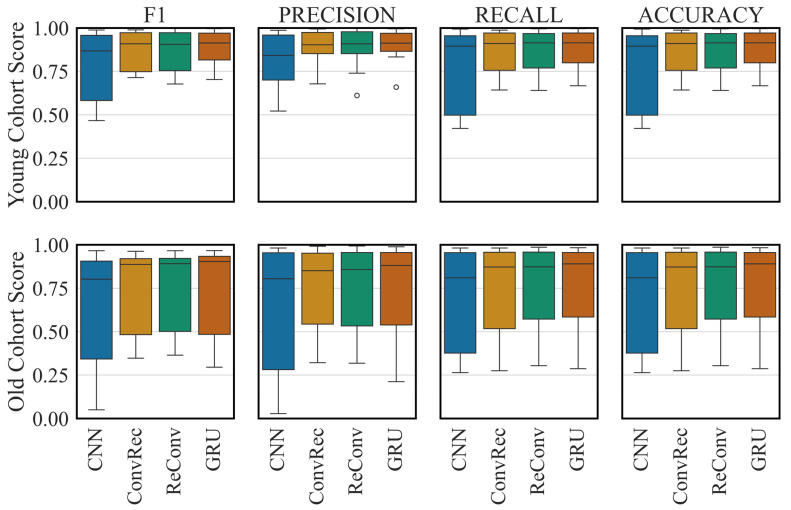
The distributions of F1-score, precision, recall and accuracy on the Young and Old Cohort Datasets for different architectures. CNN: convolutional neural network. GRU: gated recurrent unit. ConvRec: CNN followed by GRU. ReConv: GRU followed by CNN. Points shown beyond the whiskers represent statistical outliers inherent to the boxplot visualisation.

The recurrent architecture, GRU, achieved the highest average (0.89 ± 0.10) and weighted (0.95 ± 0.05) F1-score among the architectures (Figure 1 and Figure 2), with the differences being statistically significant (*p* < 0.05). The best-performing architecture identified through hyperparameter optimisation consisted of a two-block bidirectional GRU network: the first block contains two stacked layers with 128 hidden units, followed by a second layer with also 128 neurons, with dropout (0.3) applied between recurrent layers. On the other hand, CNN resulted in the worst predictive performance (average F1-score: 0.79 ± 0.20, weighted F1-score: 0.92 ± 0.09). Combining RNNs with CNNs did not significantly affect the performance of the recurrent architecture. Showing results that were slightly better than others, ConvRec’s performance was the closest to the RNN. While all architectures performed equally well for the common activity types (walking, running, standing, sitting, lying) with an F1-score over 0.85, activities involving stairs and cycling while standing up achieved the lowest scores. RNN still outperformed others with the lowest F1-score of 0.70 for the stair descent.

Overall, the models did not exhibit strong signs of global overfitting, as they maintained good performance on the Old Cohort Dataset despite being trained solely on the Young Cohort Dataset. RNN had the best performance (F1-score, average: 0.73 ± 0.29, weighted: 0.93 ± 0.05) followed by the ReConv (F1-score, average: 0.74 ± 0.26, weighted: 0.92 ± 0.05). While the common activities achieved an F1-score over 0.90, the stair ascent and descent tasks could be best predicted by the ReConv (weighted F1-score: 0.38 and 0.37, respectively) improving the performance of RNN for these tasks (0.30 and 0.35, respectively).

### 3.2. Sampling Frequency, Sensor Locations and Window Length

**Figure 3:**
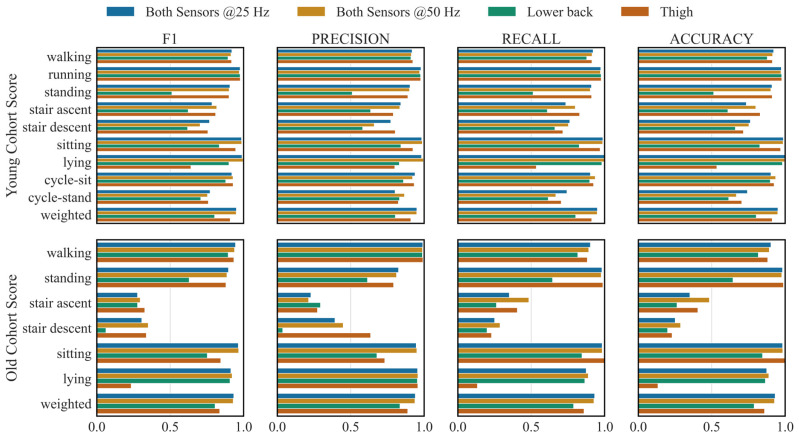
F1-score, precision, recall and accuracy on the Young and Old Cohort Datasets for different experiments (sampling frequency and sensor location). Neural network architecture and hyperparameters were optimised for each experiment. The metrics per activity and their average weighted by their frequency (“weighted”) are presented.

**Figure 4:**
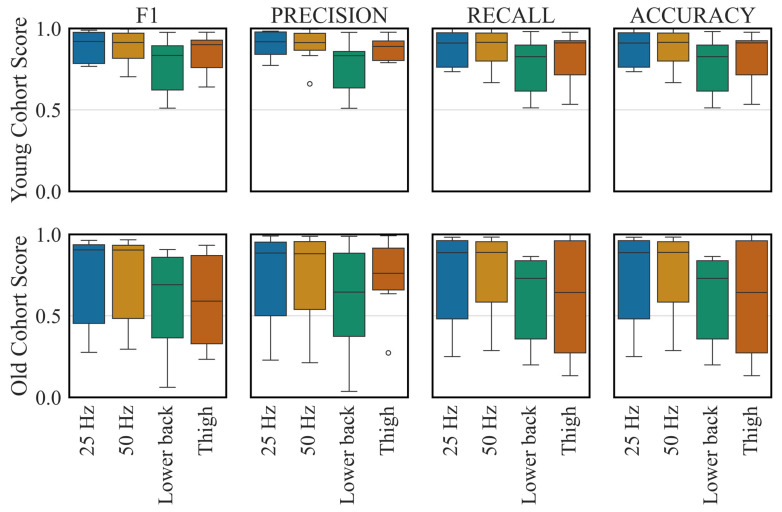
The distributions of F1-score, precision, recall and accuracy on the Young and Old Cohort Datasets for different experiments (sampling frequency and sensor location). Neural network architecture and hyperparameters were optimised for each experiment. Points shown beyond the whiskers represent statistical outliers inherent to the boxplot visualisation.

RNN still outperformed other architectures with downsampled data and in different sensor locations (Figure 3 and Figure 4). Downsampling across sampling frequencies (50, 25, 20, 15, and 10 Hz) had no statistically significant effect on overall performance (*p* > 0.05). Weighted F1-scores in the LOPO-CV evaluation remained close to 0.95 across all frequencies (Figures S1 and S2), and generalisation performance on the old cohort was similarly stable, with weighted F1-scores ranging from 0.94 ± 0.05 (15 Hz) to 0.93 ± 0.05 (all other frequencies). The activities most affected by changes in sampling frequency were stair ascent and descent. For example, reducing the sampling frequency from 50 Hz to 25 Hz degraded stair ascent performance by 4%, while stair descent performance improved by 10%. In contrast, when downsampling from 50 Hz to 10 Hz, recognition performance on the old cohort for stair ascent and descent decreased by 16% and 72%, respectively.

While using both sensors yielded the best performance in the LOPO-CV evaluation (Figure 3 and Figure 4), the thigh sensor alone achieved significantly higher performance than the lower back sensor alone (weighted F1-score: 0.91 ± 0.08 vs. 0.80 ± 0.14, *p* < 0.05). Standing, stair ascent and descent predictions were highly dependent on the thigh accelerometer. Thigh accelerometer or both accelerometers predicted the standing activity with F1-score of 0.90 unlike the lower back sensor alone which had only F1-score of 0.51. Sitting and lying predictions were improved by having the two sensors together. The network’s performance on the old cohort was remarkably improved by the two accelerometers compared to the single sensor configurations (weighted F1-score: 0.81 ± 0.12, 0.84 ± 0.20 and 0.93 ± 0.05 for lower back, thigh and combined). Prediction of sitting by the combined sensors improved to 0.97 F1-score from 0.84 for thigh and 0.75 for lower back sensor.

The effect of window length was negligible, without compromising performance. The performance of the RNN processing both sensors at 50 Hz using the 200-frames and 3000-frames of windows were practically the same (weighted F1-score: 0.94 ± 0.05 in both cases, *p* > 0.05).

### 3.3. A Generalizable Neural Network by Combining Datasets

**Figure 5:**
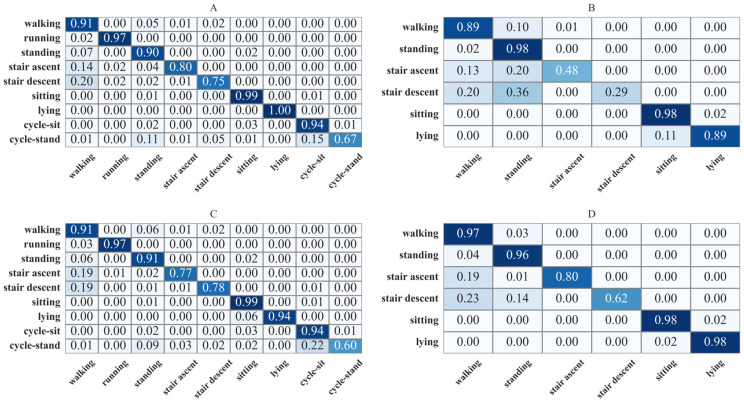
Confusion matrix for predictions on datasets, where darker shades indicate higher values. (**A**): Trained on the Young, tested on the Young Cohort Dataset. (**B**): Trained on the Young, tested on the Old Cohort Dataset. (**C**): Trained on the combined, tested on the Young Cohort Dataset. (**D**): Trained on the combined, tested on the Old Cohort Dataset.

The RNN with tuned hyperparameters was trained using both sensors sampled at 50 Hz (Figure 5, Figures S3 and S4). When trained and tested on the young cohort (Figure 5A), performance was high across activities, with recall values of 0.91 (walking), 0.97 (running), 0.90 (standing), 0.80 (stair ascent) and 0.75 (stair descent). Applying this model to the old cohort (Figure 5B) revealed reduced performance for stair ascent and descent (recall 0.48 and 0.29), with frequent confusion with standing (20% and 36%) and walking (13% and 20%), while other activities remained robust (e.g., walking 0.89, sitting 0.98). Training on the combined dataset preserved performance on the young cohort (Figure 5C; weighted F1-score: 0.94 ± 0.04, *p* > 0.05) and substantially improved performance on the old cohort (Figure 5D; weighted F1-score: 0.97 ± 0.02, *p* < 0.05), with recall for stair ascent and descent increasing to 0.80 and 0.62, respectively, and reduced misclassification into walking (19% and 23%).

## 4. Discussion

Our findings showed that the recurrent architecture achieved the best overall performance; however, the hybrid architectures (ReConv and ConvRec) also performed competitively and achieved comparable performance for several activities. Sampling frequency did not practically affect prediction performance. In contrast, sensor location influenced performance, with the thigh sensor outperforming the lower back sensor, and the combined configuration yielding the best results. The inclusion of data from the old cohort in the training phase improved the performance.

### 4.1. Neural Network Architecture Validation on the Young Cohort Dataset and Generalizability to Old Cohort Dataset

The neural network architecture emerged as the most important factor in the predictive performance. The recurrent network, GRU, effectively learned hidden features to predict activities at each time step and outperformed prior work on the same dataset using LOPO-CV [35] with higher accuracy (0.81 ± 0.18 vs. 0.89 ± 0.10). However, some activities, including stair ascent and descent, remained a persistent challenge for the current modelling approach. On the old cohort, our model also outperformed Ustad, Logacjov [34], who merged stair ascent and descent into walking, whereas our approach treated them separately. The discrepancy between the two evaluation schemas was expected, given mobility differences between cohorts, with some old participants using walking aids. Unlike their approach, which predicted a single activity for each 5 s window (250 frames), our method captured more fine-grained activity changes. Furthermore, the impact of data window length in model performance evaluation was minimal. The networks trained on 200-frame windows maintained comparable performance even when tested with much longer windows. For instance, testing with a 10 min window (vs. 4 s training window) revealed only minor trade-offs between activities (e.g., improvements in stair tasks but slight decline in cycling tasks), while overall metrics remained stable, with improved computational efficiency at longer windows. Despite GRU’s superior performance, combining it with CNNs (tested in different configurations, including CNN followed by RNN, RNN followed by CNN and multi-head CNNs) did not improve results. GRU remained the best architecture for stair ascent and descent in LOPO-CV, while in the old cohort these activities were predicted slightly better by ReConv.

Analysing per-activity performance, the GRU confusion matrix showed explainable and reasonable patterns. First, the lowest accuracy in LOPO-CV occurred for cycle-stand, which was almost equally misclassified as standing and cycle-sit. This confusion was reasonable given the overlap in postures as the activity names imply. Second, stair ascent and descent were misclassified as walking (LOPO-CV: 13% and Old Cohort: 20%). In the old cohort, 20% of stair ascent and 36% of descent were misclassified as standing, likely due to the fact that old people tend to perform these tasks significantly slower than young people due to stair negotiation [36,37]. Similarly, 10% of walking in old participants was classified as standing, likely because very slow walking approximates a standing posture, consistent with the age-related decline in gait speed [38].

A small number of predictions did not belong to the old cohort’s label set, which is inevitable given the predefined activity classes. These cases were rare, with only 0.003% and 0.08% of the dataset predicted as running and cycle-sit, respectively.

### 4.2. Sampling Frequency and Sensor Locations

Downsampling did not affect overall network performance on either dataset with only a reduction observed at 10 Hz for stair ascent and descent. These findings are consistent with prior work suggesting that sampling frequencies around 20 Hz are sufficient for wearable activity recognition [39] and that higher frequencies do not improve accuracy beyond this threshold [40]. However, more aggressive downsampling reduces the temporal resolution of the signal, which may limit the ability to capture rapid transitions and fine-grained temporal dynamics. In the context of frame-level activity recognition, this effectively shifts the problem closer to a window-based prediction regime. Therefore, while sampling frequencies of approximately 15–20 Hz appear sufficient for maintaining overall performance, higher frequencies may still be preferable when fine temporal resolution is required. Additionally, using both sensors as inputs yielded better results than using a single sensor, as expected due to increased information content for neural networks to exploit. Between the single-sensor models, the thigh sensor outperformed the lower back sensor overall, though performance varied by activity, consistent with previous work [41]. This difference is biomechanically plausible. The thigh undergoes pronounced, activity-specific movement patterns during locomotor tasks such as walking, running, and stair ascent/descent, resulting in clearer and more discriminative acceleration signatures. In contrast, acceleration signals measured at the lower back predominantly reflect trunk motion, which is more attenuated and often more similar across different activities. The largest discrepancies were observed for standing when using only the lower back sensor and for lying when using only the thigh sensor. This can be explained by the fact that the lower back sensor maintains a similar orientation during standing and sitting, whereas the thigh sensor exhibits similar orientations during sitting and lying. Consequently, the reduced signal contrast at each location for these activity pairs limits class separability and increases confusion when only a single sensor is used. This confusion in single-sensor models could potentially be reduced by labelling transitional movements, as transitions contain information about both the preceding and following activities that the network can exploit, even though recognising transitions remains inherently challenging [42].

### 4.3. A Generalizable Neural Network by Combining Datasets

Combining the two datasets resulted in the best predictive performance on the old cohort. This suggested that inclusion of data from people with different mobility characteristics is required to achieve satisfactory recognition performance [34]. The networks, serving as general or population-wide recognition models, can be personalised for each individual [43] through fine-tuning, which involves additional training using the individual’s data.

### 4.4. Limitations

This study has several limitations. First, the data collection procedure involved predefined activity protocols, which may have reduced the ecological validity of natural behaviour. Second, the range of neural architectures evaluated in this study was limited, and other architectures (e.g., 2D CNNs, transformers) were not investigated, which may have influenced the observed performance. Furthermore, there was a tendency of the networks to predict more frequent activities, which is a common problem in imbalanced datasets [44]. Considering the confusion matrices, our networks were not strongly affected; however, this issue could be further mitigated by oversampling less frequent labels while adding slight perturbations to the duplicated sensor data to avoid overfitting. This issue could also be handled by data augmentation techniques ranging from time-based numerical approaches [45] to more sophisticated methods such as generative adversarial networks and diffusion models [46,47,48].

## 5. Conclusions

Overall, this study demonstrated that RNNs are well-suited for HAR using accelerometers in the context of granular activity recognition, likely thanks to their ability to model longer-range temporal dependencies. The trained networks provided accurate, fine-grained predictions at the frame-level, capturing subtle transitions between activities while remaining robust to variations in window length. Including data from the old cohort during training improved generalizability by capturing greater population diversity. From a practical perspective, these findings are important for real-world deployment. The observation that performance was maintained at lower frequencies, and that acceptable accuracy can be achieved using a single thigh-mounted sensor, supports the feasibility of low-power, minimally intrusive wearable systems using the relatively lightweight RNN architectures evaluated in this study, particularly when compared with the higher computational demands typically associated with recent architectures. This has direct implications for long-term adherence and acceptability, particularly in old cohorts and healthcare populations. Overall, the presented approach is a promising step to continuous health monitoring using low-cost, lightweight wearable sensors and neural networks to process their data.

## Data Availability

The datasets used in the study are openly available in GitHub at https://github.com/ntnu-ai-lab/harth-ml-experiments (accessed on 12 January 2026).

## Supplementary Materials

The following supporting information can be downloaded at https://www.mdpi.com/article/10.3390/s26041320/s1. Figure S1. F1-score, precision, recall and accuracy on the Young and Old Cohort Datasets for multiple sampling frequencies. Neural network architecture and hyperparameters were optimised for each experiment. The metrics per activity and their average weighted by their frequency (“weighted”) are presented. Figure S2. The distributions of F1-score, precision, recall and accuracy on the Young and Old Cohort Datasets for multiple sampling frequencies. Neural network architecture and hyperparameters were optimised for each experiment. Points shown beyond the whiskers represent statistical outliers inherent to the boxplot visualisation. Figure S3. F1-score, precision, recall and accuracy on the Young and Old Cohort Datasets for neural networks trained solely on the Young Cohort Dataset and the Combined Dataset. The metrics per activity and their average weighted by their frequency (“weighted”) are presented. Figure S4. The distributions of F1-score, precision, recall and accuracy on the Young and Old Cohort Datasets for neural networks trained solely on the Young Cohort Dataset and the Combined Dataset. The metrics per activity and their average weighted by their frequency (“weighted”) are presented. Points shown beyond the whiskers represent statistical outliers inherent to the boxplot visualisation.

## Abbreviations

The following abbreviations are used in this manuscript:

HAR	Human activity recognition
CNN	Convolutional neural network
RNN	Recurrent neural network
GRU	Gated recurrent unit
ConvRec	Convolutional–Recurrent network
ReConv	Recurrent–Convolutional network
LOPO	Leave-one-participant-out
