# Supplementary material for: Neural Network-Based Granular Activity Recognition from Accelerometers: Assessing Generalizability Across Diverse Mobility Profiles
Source: Sensors (Basel). 2026 Feb 18;26(4):1320. doi: 10.3390/s26041320 (PMC12944021; doi:10.3390/s26041320)
Supplement: Supplementary file 1 [file sensors-26-01320-s001.zip › sensors-4112961-supplementary.pdf]

Supplementary Materials

Neural Network-Based Granular Activity Recognition from Accelerometers: Assessing Generalizability Across Diverse Mobility Profiles

Metin Bicer [1,2], James Pope [3], Lynn Rochester [1,2], Silvia Del Din [1,2,*], Lisa Alcock [1,2]

[1] Translational and Clinical Research Institute, Faculty of Medical Sciences, Newcastle University, Newcastle upon Tyne, UK
[2] NIHR Newcastle Biomedical Research Centre, Newcastle University and The Newcastle upon Tyne Hospitals NHS Foundation Trust, Newcastle upon Tyne, UK
[3] School of Engineering Mathematics and Technology, University of Bristol, Bristol, UK
* Correspondence: silvia.del-din@newcastle.ac.uk; Tel.: +44 (0)191 2081131

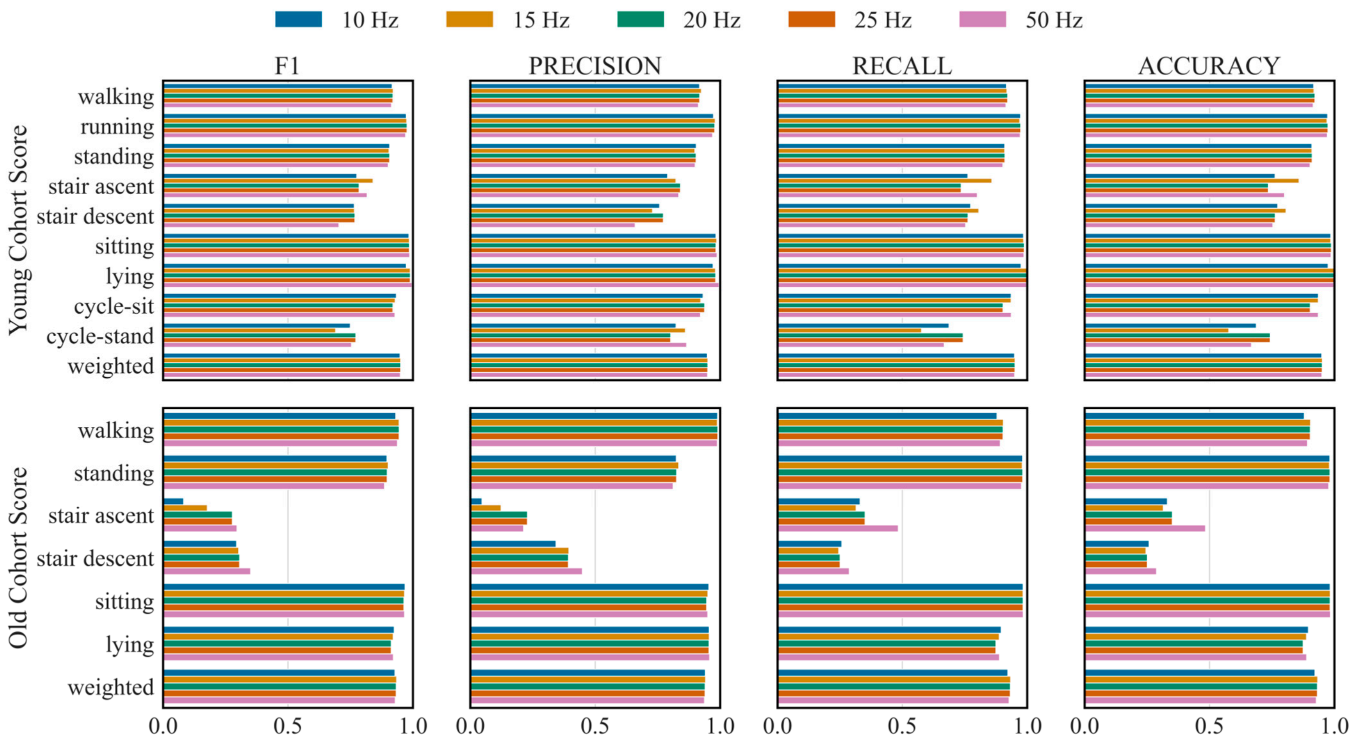

Figure S1. F1-score, precision, recall and accuracy on the Young and Old Cohort Datasets for multiple sampling frequencies. Neural network architecture and hyperparameters were optimised for each experiment. The metrics per activity and their average weighted by their frequency ("weighted") are presented.

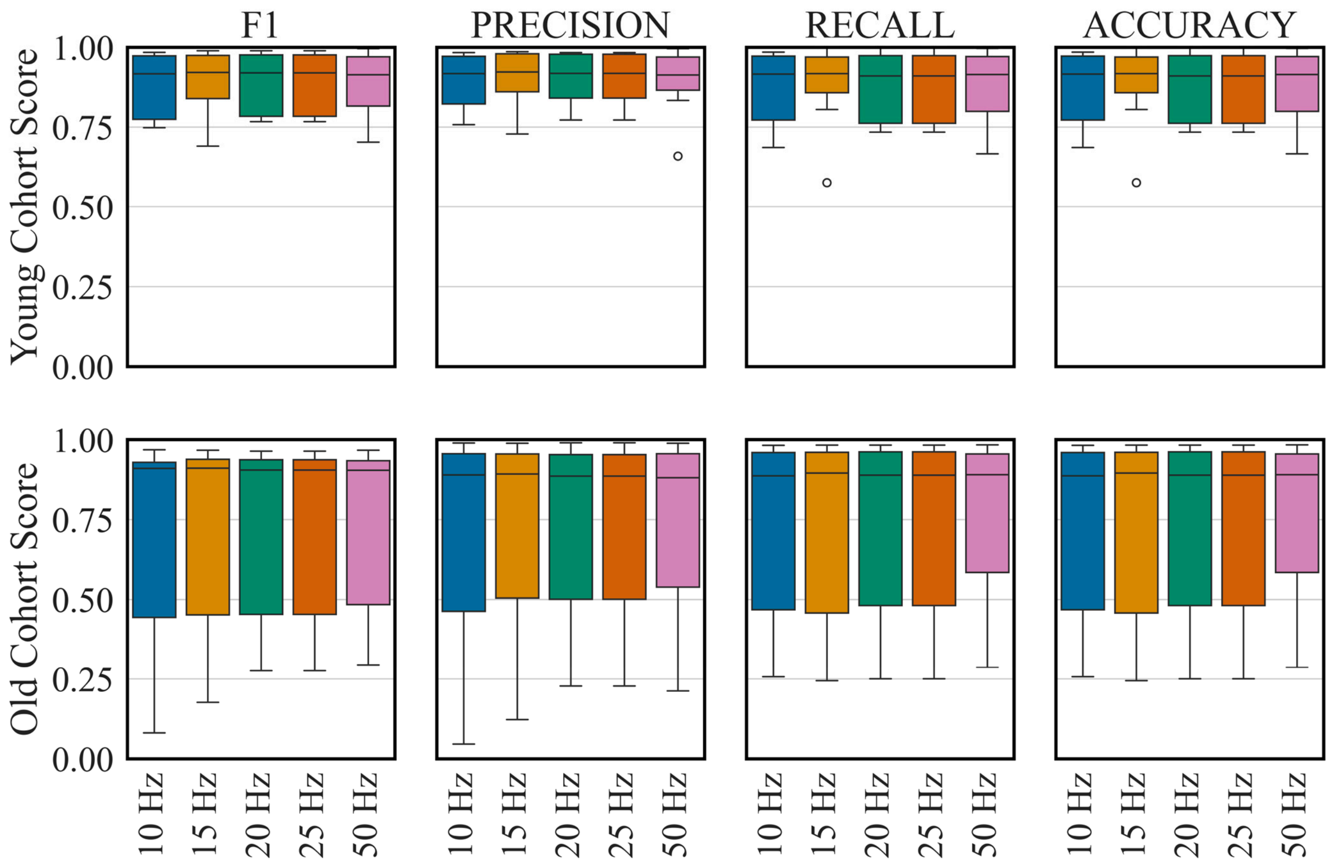

Figure S2. The distributions of F1-score, precision, recall and accuracy on the Young and Old Cohort Datasets for multiple sampling frequencies. Neural network architecture and hyperparameters were optimised for each experiment. Points shown beyond the whiskers represent statistical outliers inherent to the boxplot visualisation.

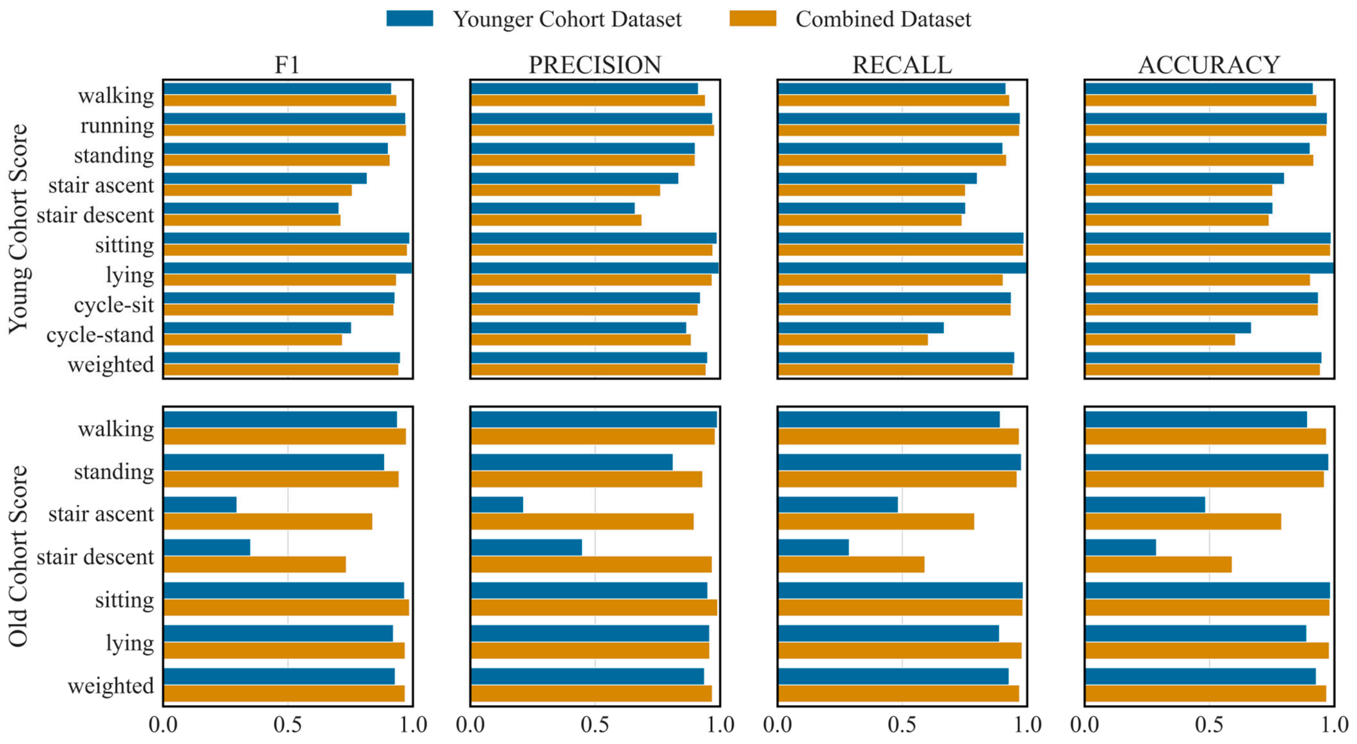

Figure S3. F1-score, precision, recall and accuracy on the Young and Old Cohort Datasets for neural networks trained solely on the Young Cohort Dataset and the Combined Dataset. The metrics per activity and their average weighted by their frequency ("weighted") are presented.

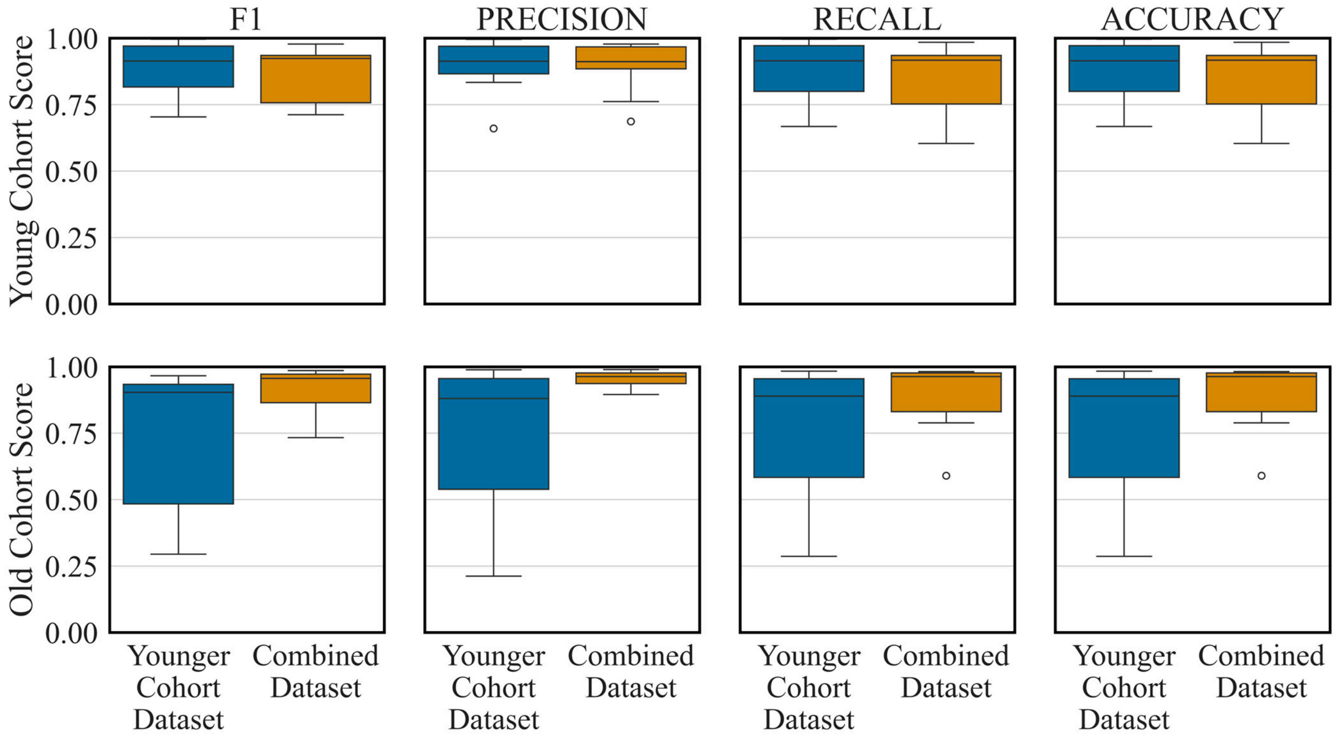

Figure S4. The distributions of F1-score, precision, recall and accuracy on the Young and Old Cohort Datasets for neural networks trained solely on the Young Cohort Dataset and the Combined Dataset. The metrics per activity and their average weighted by their frequency ("weighted") are presented. Points shown beyond the whiskers represent statistical outliers inherent to the boxplot visualisation.
